# *Nm*Pin from the marine thaumarchaeote *Nitrosopumilus maritimus* is an active membrane associated prolyl isomerase

**DOI:** 10.1186/s12915-016-0274-1

**Published:** 2016-06-27

**Authors:** Lukas Hoppstock, Franziska Trusch, Christoph Lederer, Pieter van West, Martin Koenneke, Peter Bayer

**Affiliations:** Department of Structural and Medicinal Biochemistry, Centre for Medical Biotechnology, University of Duisburg-Essen, Universitätsstr. 1-4, 45141 Essen, Germany; Aberdeen Oomycetes Laboratory, Institute of Medical Sciences, University of Aberdeen, Foresterhill, AB25 2ZD Aberdeen, UK; Organic Geochemistry Group, MARUM Center for Marine Environmental Sciences, University of Bremen, Leobener Str. MARUM, 28359 Bremen, Germany

**Keywords:** Parvulin, *Nm*Pin, *Nitrosopumilus maritimus*, Thaumarchaeota, Archaea, PPIase, Isomerase, Membrane

## Abstract

**Background:**

Peptidyl-prolyl isomerases (PPIases) are present in all forms of life and play a crucial role in protein folding and regulation. They catalyze the *cis-trans* isomerization of the peptide bond that precedes proline residues in numerous proteins. The parvulins, which is one family of PPIases, have been extensively investigated in several eukaryotes. However, nothing is known about their expression, function and localization in archaea.

**Results:**

Here, we describe the endogenous expression, molecular structure, function and cellular localization of *Nm*Pin, a single-domain parvulin-type PPIase from *Nitrosopumilus maritimus.* This marine chemolithoautotrophic archaeon belongs to the globally abundant phylum Thaumarchaeota. Using high resolution NMR spectroscopy we demonstrate that the 3D structure of *Nm*Pin adopts a parvulin fold and confirmed its peptidyl-prolyl isomerase activity by protease-coupled assays and mutagenesis studies. A detailed topological analysis revealed a positively charged lysine-rich patch on the protein surface, which is conserved in all known parvulin sequences of thaumarchaeotes and targets *Nm*Pin to lipids in vitro*.* Immunofluorescence microscopy confirms that the protein is attached to the outer archaeal cell membrane in vivo*.* Transmission electron microscopy uncovered that *Nm*Pin has a uniform distribution at the membrane surface, which is correlated with a native cell shape of the prokaryote.

**Conclusion:**

We present a novel solution structure of a catalytically active thaumarchaeal parvulin. Our results reveal that a lysine-rich patch in *Nm*Pin mediates membrane localization. These findings provide a model whereby *Nm*Pin is located between the archaeal membrane and the surface layer and hence suggest proteins of the S-layer as the key target substrates of this parvulin.

**Electronic supplementary material:**

The online version of this article (doi:10.1186/s12915-016-0274-1) contains supplementary material, which is available to authorized users.

## Background

Proteins are biomolecules acting as scaffolds, signal transmitters or catalysts of chemical reactions in living cells. Before they can commence their tasks they need to undergo intensive folding steps to adopt their proper three-dimensional topologies. The *cis-trans* isomerization of peptide bonds in Xaa-Pro moieties (Xaa being any amino acid) is essential but also a rate-limiting step in such protein folding processes [1]. Due to a high energy barrier (~20 kcal/mol) between the two almost isoenergetic conformers, the rate of interconversion is extremely slow [1, 2]. However, an important group of proteins, called the peptidyl-prolyl *cis-trans* isomerases (PPIases), catalyze and accelerate this reaction and thereby essentially control the folding of proteins [3, 4]. PPIases are grouped in three classes – the cyclophilins (CYP) [5], FK506-binding proteins (FKBP) [6], and the parvulins [7] – according to their topology [8].

Parvulins, a group of small globular proteins with a distinctive βα_3_βαβ_2_-fold, are found in all kingdoms of life [9, 10]. By far the most well-studied parvulin is the human Pin1, a phosphorylation-dependent molecular switch, which is involved in cell cycle and transcriptional regulation as well as protein quality control [11–14]. Pin1 is reported to influence ageing, cancer development and neurodegenerative processes in Alzheimer’s and Parkinson’s diseases (reviewed in [15]). Prokaryotic parvulins, such as the structurally characterized SurA [16–19] and PpiD [20, 21] from *Escherichia coli*, PrsA from *Bacillus subtilis* [22–24] or PrsA from *Staphylococcus aureus* [25], are involved in folding and maturation of extracellular, periplasmic and outer membrane proteins. In contrast to eukaryotic Pin-type parvulins found in yeast, metazoans and multicellular archaeplastidae, the prokaryotic representatives lack a recognition site for phosphorylated target residues [20, 25–27].

Except for the smallest member and archetype of the parvulin family, Par10 from *E. coli* [7, 9, 27], all proteins mentioned above contain N- or C-terminal extensions/domains in addition to the parvulin domain. Functional studies have shown that the parvulin domain of PpiD and the N-terminal domain of SurA both lack *cis-trans* isomerase activity, but possess chaperone activity [20, 28]. Due to their function, some parvulins are tightly linked to membranes: PrsA, a foldase for secreted proteins and essential for cell wall assembly in *B. subtilis* is connected with a lipid-anchor at an N-terminal cysteine residue to the outer leaflet of the cell membrane [22, 29, 30] and PpiD, the periplasmic foldase of outer membrane proteins, is embedded in the lipid double-layer via an N-terminal transmembrane helix [31]. The PrsA of *L. monocytogenes* is also attached to the membrane by a lipid-anchor and supports the correct folding of secreted proteins during infection and hence plays an important role for the virulence of the pathogen [32].

During the last decade, the number of sequenced and annotated archaeal genomes has increased, with some of them including parvulin homologue genes [33]. In contrast to the multi-domain parvulins described above, all identified archaeal parvulins consist of a single domain (sdPar) [34] and exhibit strong homologies to Par10 [27]. The first and only structurally characterized archaeal parvulin *Cs*PinA [26] originates from the psychrophilic thaumarchaeote *Cenarchaeum symbiosum*, which lives as a symbiont of the marine sponge *Axinella mexicana* and therefore eludes pure cultivation. Hence, its expression has not been demonstrated in vivo and no further studies regarding the localization or the cellular role of *Cs*PinA have been performed. Only the 3D structure of the archaeal representative *Cs*PinA has been characterized after expressing the protein recombinantly in *E. coli.* Furthermore, neither data of its catalytic activity nor its substrate specificity are available so far.

More recently, Könneke et al. [35] reported the isolation of the first thaumarchaeote into pure culture. *Nitrosopumilus maritimus* is 0.17–0.22 μm in diameter and 0.5–0.9 μm in length and grows chemoautotrophically by oxidizing ammonia to nitrite and by fixing carbon dioxide as a sole carbon source. Due to their ubiquity and high abundance, ammonia-oxidizing thaumarchaeotes have become recognized as major nitrifiers in a wide range of habitats [36]. Here, we provide novel insight into the cellular localization of the endogenous parvulin *Nm*Pin in *N. maritimus* and present a detailed high resolution structure. *Nm*Pin turned out to be a catalytically active prolyl-isomerase with a parvulin-type fold that is associated to the archaeal cell membrane.

## Results

### *Nm*Pin is endogenously expressed and is a catalytically active sdPar

**Fig. 1:**
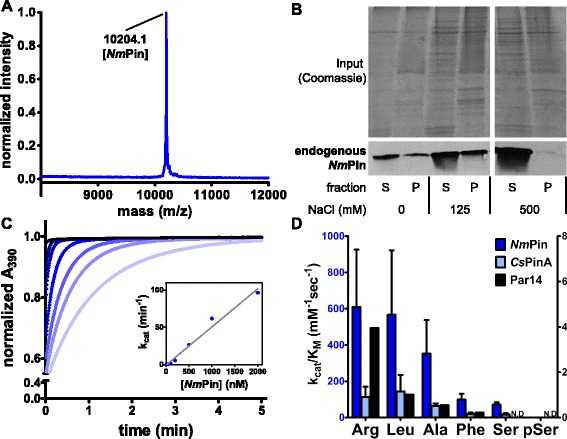
Expression of endogenous protein and biochemical characterization of recombinant *Nm*Pin. **a** MALDI-TOF spectrum of recombinant *Nm*Pin. **b** Upper illustration, representative SDS-PAGE gel of *N. maritimu*s cell lysates (Coomassie staining). Cells were treated with different salt concentrations before fractionation. Lower illustration, single Western blot experiment of endogenous *Nm*Pin using α-*Nm*Pin antibody. The corresponding fractions and salt conditions are annotated below. **c** Absorption curves revealing the time resolved cleavage of the peptide Suc-A-R-P-F-pNA in the presence of different *Nm*Pin concentrations in a protease-coupled isomerase assay. The amount of *Nm*Pin added to the reaction is indicated by a color gradient from 0 μM (bright blue) to 2 μM (dark blue). A steeper initial slope represents a faster isomerization. Insert: plot of catalytic constants k_cat_ derived from absorption curves by bi-exponential curve fits against the corresponding *Nm*Pin concentrations. **d** Diagram of k_cat_/K_M_ values for various substrates comprising the scaffold Suc-A-X-P-F-pNA measured for *Nm*Pin (blue, left scale), CsPinA (light blue, left scale) and Par14 (black, right scale) [4]. The residue X is specified on the x-axis of the diagram. Data are presented as means ± standard deviation from six (two for *Cs*Pin) independent measurements with different enzyme concentrations

In the genome of *N. maritimus* we identified an open reading frame, which encodes a putative 91 amino acid parvulin-like PPIase [34]. The putative parvulin, which we named *Nm*Pin, was recombinantly produced. The expected molecular mass and purity were confirmed by mass spectrometry (Fig. 1a), using α-*Nm*Pin, an antibody that we generated to analyze the expression of endogenous *Nm*Pin by western blot under different osmotic conditions, as the salt concentration may be critical for the marine organism. For all conditions the blots showed a distinctive band at ~10 kDa indicating the expression of *Nm*Pin as a single domain parvulin (sdPar) also in vivo. At low salt concentrations, *Nm*Pin was found in the supernatant as well as in the membrane enriched pellet fraction of lysed *N. maritimus*. In contrast, high salt levels up to 500 mM NaCl disturbed the attachment to the membrane fraction and *Nm*Pin was predominantly found in the supernatant of *N. maritimus* lysates (Fig. 1b). Although this is, to our knowledge, the first description of an endogenous expression of an archaeal parvulin-type PPIase, the amounts of proteins were not sufficient for further biochemical and biophysical studies. Thus, we examined the catalytic activity of recombinant *Nm*Pin and performed a protease-coupled isomerase assay. In the presence of increasing concentrations of *Nm*Pin an accelerated interconversion of the peptide Suc-A-R-P-F-*p*NA from *cis* to *trans* isoform was observed (Fig. 1c). We additionally assayed the substrate selectivity and specificity of *Nm*Pin by alternating the residue preceding the proline in the model substrate peptides. The fastest PPIase activity was observed for peptides carrying an arginine or a hydrophobic branched leucine at this position (Fig. 1d) with a rate constant for catalytic efficiency of *k*_*cat*_*/K*_*M*_ = 6.1 ± 1.3 × 10^5^ M^–1^s^–1^ and *k*_*cat*_*/K*_*M*_ = 5.7 ± 1.5 × 10^5^ M^–1^s^–1^, respectively. As expected from the high homology, the same substrate selectivity was observed for *Cs*PinA. While the human Par14 shows the same substrate selectivity the bacterial *Ec*Par10 from *Escherichia coli* prefers Leu to Phe and Ala [7]. In comparison to *Nm*Pin, the catalytic efficiency of *Ec*Par10 is 30 times higher (K_cat_/K_M_ = 1.69 × 10^7^ M^–1^s^–1^), while the one for Par14 is about 600 times lower for a Leu-Pro model substrate. Similar to *Ec*Par10 and Par14 but in contrast to the eukaryotic homologs ESS1 (*C. albicans*) and Pin1 (human) [12, 36–38], archaeal PPIases do not catalyze the isomerization of a phosphorylated serine motif (Fig. 1d).

### The solution structure of *Nm*Pin shows a parvulin-fold and exhibits a novel lysine-rich patch

**Fig. 2:**
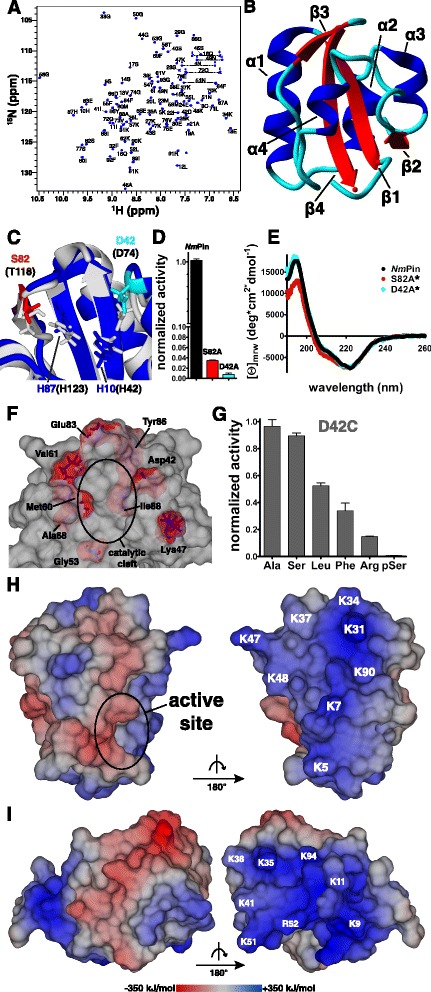
Solution structure and structural features of the parvulin *Nm*Pin from *Nitrosopumilus maritimus*. **a** Assigned ^1^H-^15^N-HSQC of recombinant *Nm*Pin. Assignment was performed with CcpNmr analysis using HNCACB/CBCACONH spectra to trace the protein sequence chain. **b** Ribbon presentation of the tertiary structure of *Nm*Pin. The parvulin fold comprises a central four-stranded β-sheet (red) surrounded by four α-helices (blue), which are connected through short loops and turns (cyan). Secondary structure elements are annotated with Greek letters. **c** Superposition of the structures of *Nm*Pin (blue) and Par14 (grey, PDB ID: 3UI4) with the two histidines (dual histidine motive [41]) in the respective leading color. The outer residues of the catalytic tetrad of *Nm*Pin (D42 in cyan, S82 in red) and of hPar14 (D74 and T118 in brackets) [40] are indicated. **d** A diagram of normalized isomerase activity measured for wildtype *Nm*Pin (black column) and proteins comprising either S82A (red column) or D42A (cyan column) single residue mutations within the catalytic tetrad. The mutants S82A and D42A retain only a low residual activity of 3.5 % and 0.7 %, respectively. Data were recorded in duplicates and are presented as means ± standard deviation. **e** Circular dichroism spectra of *Nm*Pin (black), *Nm*Pin_D42A_ (cyan) and *Nm*Pin_S82A_ (red) shown as mean residue ellipticity (mrw). *Datasets were normalized to the wildtype spectrum for better comparison of the three protein folds. **f** Surface representation of *Nm*Pin. Residues involved in substrate binding are mapped on the molecular surface (red, side chain atoms in purple sticks). Residues were derived from chemical shift perturbation analysis in a ^1^H-^15^N-SOFAST-HMQC titration experiment using Suc-A-R-P-F-pNA as a ligand. **g** Diagram of the normalized activity for various substrates comprising the scaffold Suc-A-X-P-F-pNA measured for *Nm*Pin_D42C_. The residue X is specified on the x-axis of the diagram. Data are normalized to the substrate with the highest activity and are presented as means ± standard deviation from two independent measurements with different enzyme concentrations. **h** and **i** Electrostatic potential of the molecular surface of *Nm*Pin and *Cs*Pin calculated with YASARA [83] using the Particle Mesh Ewald approach. The intensity of the surface potential is gradually colored from dark red (negative) over grey (neutral) to dark blue (positive) representing energy levels from –350 to +350 kJ/mol. The active site of *Nm*Pin is encircled. The opposite sites of both *Nm*Pin as well as *Cs*Pin are defined by a positively charged lysine-rich patch as labelled, respectively.

The three-dimensional structure of recombinant *Nm*Pin was determined by high resolution nuclear magnetic resonance (NMR) spectroscopy (Table 1, Fig. 2a).

**Table 1:** NMR and refinement statistics for *Nm*Pin (residue 4–93)

NOE-based distance restraints	
Intra-residual	352
Sequential	436
Medium range (2 ≤ Ii-jI ≤ 4)	318
Long range (Ii-jI ≥ 5)	740
Total	1846
Violated	2 Distances, 3 vdW
Other restraints	
Φ + ψ dihedral restraints	0
Hydrogen bond restraints	80
Coordinate precision (Å)	
Backbone	0.30 ± 0.04
All	0.80 ± 0.03
Whatcheck	
First-generation packing quality	2.918 ± 1.251
Second-generation packing quality	4.617 ± 1.785
Ramachandran plot appearance	0.886 ± 0.227
χ^1^/χ^2^ rotamer normality	−3.471 ± 0.486
Backbone conformation	1.647 ± 0.186
Ramachandran plot (%)	
Most favored regions	91.9
Allowed regions	7.0
Generously allowed regions	0.9
Disallowed regions	0.3

The protein adopts the typical βα_3_βαβ_2_ fold common for all parvulin proteins [8, 39] with short connecting loops and turns between the secondary structure elements (Fig. 2b). The closest homolog of *Nm*Pin is *Cs*PinA from the phylogenetically related thaumarchaeote *C. symbiosum* with 78.4 % sequence identity and a root mean square deviation (RMSD) of 1.28 Å (PDB ID: 2RQS, Tab. 2).

**Table 2:** Pairwise root mean square deviation (RMSD) of *Nm*Pin to parvulin homologs. RMSD values were calculated with YASARA using the alignment tool Mustang [84]

Structure	PDB ID	RMSD (Cα) [Å]	Aligned residues	% Seq. identity	Citation
CsPinA	2RQS	1.281	88	78.4	[26]
hPin1_PPIase	1NMW	1.301	89	44.9	[85]
SaPrsA_PPIase	2JZV	1.302	84	54.8	[25]
TbPin1_PPIase	2LJ4	1.382	89	38.2	[86]
hPar14_PPIase	3UI4	1.715	86	46.5	[40]
EcPpiD_PPIase	2KGJ	1.732	80	30.0	[20]
AtPin1	1J6Y	1.905	86	39.5	[42]
EcPar10	1JNT	2.267	84	41.7	[27]

However, regarding the folding and the special dimensions of the active site, the eukaryotic *cis-*/*trans*-isomerase Par14 from human (PDB ID: 3UI4, RMSD of 1.72 Å) shows the highest similarity to *Nm*Pin [8]. Hence, we have used the structure of Par14 to deduce and assign the catalytic tetrad of *Nm*Pin (C/D-H-H-T/S) [40]. It is built up by residues His10 and His87 (common dual histidine motif) [41], which are flanked by Asp42 and Ser82. To confirm the functionality of this derived tetrad, we mutated the two flanking residues to alanines, yielding the mutants *Nm*Pin_D42A_ and *Nm*Pin_S82A_ (Fig. 2c). The two mutant enzymes showed a significant decrease in catalysis rate when compared to the wildtype protein with residual activities of 0.7 % and 3.5 %, respectively (Fig. 2d). As the circular dichroism (CD) spectra of the mutant proteins are comparable with the wild type spectrum (Fig. 2e), structural changes as the reason for the loss of PPIase activity can be excluded. To localize the substrate binding pocket on *Nm*Pin a ^1^H-^15^N-HSQC-based NMR chemical shift perturbation experiment was performed using the -R-P- substrate peptide as a ligand. HN resonances comprising shift differences ≥ 0.04 ppm (D42, K47, G53, M60, V61, A68, E83, Y86, I88) exclusively belong to residues of the active site or to surrounding amino acids (Fig. 2f). This strongly suggests that substrates are bound and converted within the proposed catalytic groove. In addition, we have used a D42C mutant of *Nm*Pin to gain further information about the recognition of the residues N-terminal to the proline in the substrate (Fig. 2g). The *Nm*Pin_D42C_ has the same catalytic efficiency as wildtype *Nm*Pin (data not shown), but prefers short amino acids (Ala, Ser) compared to the wildtype (Arg, Leu). Interestingly, opposite to the catalytic cleft is a highly positively charged surface motif composed of eight lysines (K5, K7, K31, K34, K37, K47, K48, K90) (Fig. 2h). This prominent feature (K9, K11, K35, K38, K41, K51, R52, K94) is also found in *Cs*PinA (Fig. 2i).

### *Nm*Pin binds to lipids and is located at the outer membrane side of *N. maritimus*

**Fig. 3:**
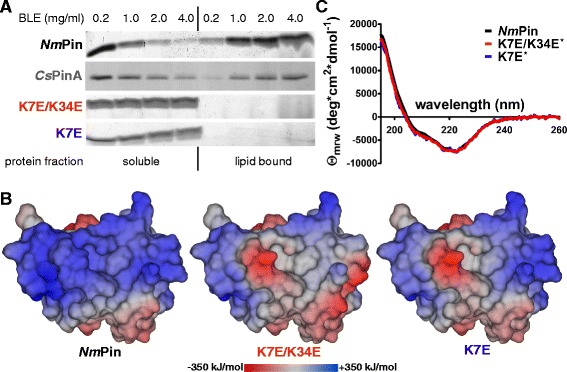
Lipid binding ability of recombinant *Nm*Pin. **a** Coomassie stained SDS-PAGE of 15 μM *Nm*Pin (wildtype, black; *Nm*Pin_K7E/K34E_, red; *Nm*Pin_K7E_, blue) as well as 15 μM *Cs*PinA (grey) in the presence of different concentrations of brain lipid extract (BLE). Layers resemble the amount of protein found in either the soluble or the lipid bound fraction after sedimentation (lipid sedimentation assay). The amounts of BLE are annotated above and the corresponding protein fractions are indicated below. **b** Electrostatic surface potential of wildtype *Nm*Pin (black), *Nm*Pin_K7E/K34E_ (red) and *Nm*Pin_K7E_ (blue) calculated with Particle Mesh Ewald approach (PME). The intensity of the potential is indicated by a color gradient from dark red (negative), over grey (neutral), to dark blue (positive) ranging from –350 to +350 kJ/mol. The mutants K7E/K34E and K7E were modeled using residue swaps in YASARA prior to calculation of the electrostatic potential. **c** Circular dichroism spectra of *Nm*Pin (black), *Nm*Pin_K7E_ (blue) and *Nm*Pin_K7E/K34E_ (red) shown as mean residue ellipticity (mrw). *Datasets were normalized to the wildtype spectrum for better comparison of the three protein folds

**Fig. 4:**
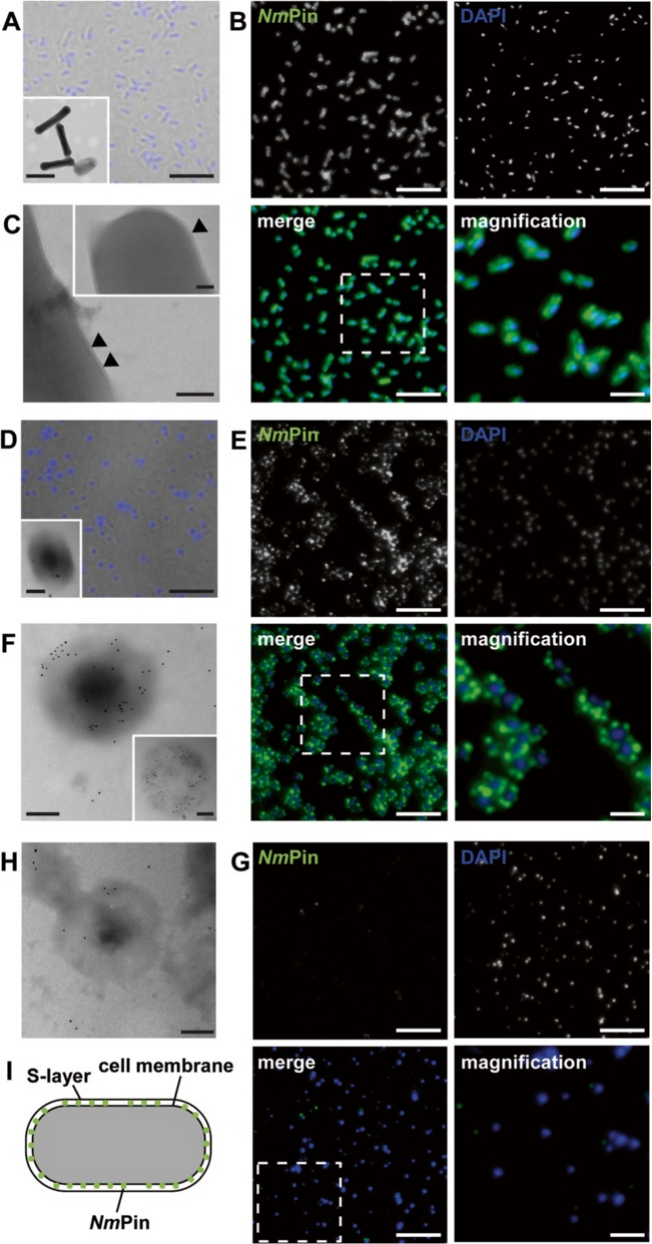
Localization of endogenous *Nm*Pin in *N. maritimus* observed by microscopy. **a** Overlay of brightfield (grey) and fluorescence (blue, DAPI stain) microscopy images (bar: 5 μm). Cells exhibit a normal rod-shaped form after paraformaldehyde (PFA) fixation before harvesting by centrifugation as observed by transmission electron microscopy (TEM) (insert, bar: 500 nm). **b** Fluorescence microscopy of PFA-fixed *N. maritimus* (bars: 5 μm)*.* Top left, immuno-staining of endogenous *Nm*Pin with Alexa488. Top right, DAPI staining of DNA. Bottom left, overlay of *Nm*Pin and DAPI stained cells. The square indicates the area of magnification. Bottom right, magnification of *Nm*Pin stained cells show a localization in the cell envelope (bar: 2 μm). **c** Detailed TEM microscopy images of PFA-fixed *N. maritimus* cells show a surface layer (S-layer) enframing the cytoplasmic membrane (arrowheads, bar: 50 nm, bar insert: 20 nm). **d** Overlay of brightfield (grey) and DAPI stain (blue) images of *N. maritimus* without PFA fixation before harvesting (bar: 5 μm). The insert shows a TEM image confirming a change in cell shape to a more spheroidal form with a very diffuse membrane structure (bar: 100 nm). **e** Fluorescence microscopy images of *N. maritimus* without PFA fixation before harvesting (bars: 5 μm). Top left, *Nm*Pin fluorescence-staining with Alexa488. Top right, DAPI stain. Bottom left, overlay of *Nm*Pin and DAPI stained cells. The square indicates the area of magnification. Bottom right image shows a magnification of the *Nm*Pin stain with a less uniform localization in the cell envelope (bar: 2 μm). **f** TEM microscopy picture of Immunogold-labelled *Nm*Pin in *N. maritimus* without PFA treatment (bars: 100 nm). Black spots representing *Nm*Pin are concentrated in the outer diffuse membrane area of *N. maritimus cells.* **g** Fluorescence microscopy images of *N. maritimus* incubated in low salt conditions (phosphate buffered saline; PBS) (bars: 5 μm). Top left, picture of immuno-stained *Nm*Pin with Alexa488, showing a significant decrease of fluorescence intensity in comparison to marine salt conditions. Top right, DAPI stained cells. Bottom left, overlay of DAPI and *Nm*Pin stained cells. The square indicates the area of magnification. Bottom right, the magnification of NmPin stain confirms the signal intensity loss (bar: 2 μm). **h** TEM microscopy image of Immunogold-labelled *Nm*Pin in *N. maritimus* after incubation in PBS (bar: 100 nm). Black spots representing *Nm*Pin are localized arbitrarily and show a detachment of *Nm*Pin from the cell envelope. **i** Schematic presentation of *Nm*Pin localization in *N. maritimus*

The wide dimension, as well as the positive charge of the lysine-rich area of *Nm*Pin, might enable and promote an interaction between the protein and negatively charged lipids of the plasma membrane. The membrane of thaumarchaeotes mainly consists of the core lipid crenarchaeol, which is a glycerol dialkyl glycerol tetraether that occurs primarily as intact polar lipids, with phosphatidic, glycosidic or phosphoglycosidic negatively charged head groups [42–46]. We hypothesize that the interaction between *Nm*Pin and the membrane is mainly based on electrostatic interactions between the positive patch on the *Nm*Pin surface and negatively charged lipid head groups. Since the production of archaeal lipids in sufficient amounts for sedimentation assays was not feasible, we have used vesicles from bovine brain extract, mainly composed of negatively charged phosphatidylinositol and phosphatidylserine, as an intact polar lipid model to study the association of recombinant *Nm*Pin to polar lipids. Measuring the association of the protein to vesicles revealed that *Nm*Pin attached to the vesicle lipids and sedimented as a protein-lipid complex (Fig. 3a). With increasing lipid concentrations (≥2 mg/mL) only limited amounts of *Nm*Pin remained in solution. Due to the conserved positively charged patch *Cs*Pin can also be sedimented by lipid vesicles. To examine if the binding of both proteins originates from Coulomb interactions of the lysine-rich area, Lys7 and Lys34 of *Nm*Pin were mutated to glutamate (K7E/K34E) and thereby partially neutralized the central positive patch (Fig. 3b). In a lipid sedimentation assay, the interaction of the mutant with the lipids was impaired and vesicle association completely abolished (Fig. 3a). The same behavior was observed for the exclusive mutation of Lys7 (Fig. 3a, b), which is highly conserved among thaumarchaeotes [34]. Structural changes due to mutations could be excluded for both constructs by performing CD spectroscopy (Fig. 3c). To substantiate that *Nm*Pin associates with the membrane, we analyzed the localization of endogenous *Nm*Pin in *N. maritimus* using fluorescence microscopy. Cells were obtained with the previously described rod-shape morphology, when fixed by the addition of paraformaldehyde (PFA) [47, 48] (Fig. 4a). After immunochemistry, a membrane-associated localization pattern was observed for endogenously expressed *Nm*Pin in *N. maritimus* (Fig. 4b). As previously shown in numerous members of the archaea [49], *N. maritimus* very likely possesses a protein-based surface layer (S-layer) on the outer side of the membrane, which can be visualized with transmission electron microscopy (TEM) as an additional envelope surrounding the cell (Fig. 4c). Hence, to confirm the in vitro lipid binding properties of recombinant *Nm*Pin and to rule out an embedding into the surface layer, the S-layer was permeabilized by eliminating the PFA fixation step before harvesting the cells. Although the cells lost their rod-shape and changed to a more spherical form with a diffuse cell membrane (Fig. 4d), endogenous *Nm*Pin was still detectable in concentrated areas at the surface of *N. maritimus* (Fig. 4e). In addition, the membrane association of *Nm*Pin was also confirmed by immunogold-labelling and subsequent TEM. Gold particles were localized in the circumference of permeabilized *N. maritimus* (Fig. 4f). Finally, *N. maritimus* was incubated in phosphate buffered saline (PBS), which on the one hand mimics low salt conditions for this marine archaea and leads to swelling and partial cell lysis, but on the other hand, the different phosphate concentrations might compete with *Nm*Pin for the binding to lipids. The fluorescence intensity, and hence the amount of endogenous *Nm*Pin, is decreased significantly under these conditions (Fig. 4g/h).

## Discussion

### *Nm*Pin is a novel functional parvulin from a marine Archaeon

In this study, we describe the endogenous expression of a novel archaeal parvulin and confirm its cellular occurrence as a single-domain protein. Our high resolution NMR structure of recombinant *Nm*Pin reveals a typical parvulin fold with all residues present to form a catalytically active site. *Nm*Pin, as well as its homologue *Cs*PinA, show isomerase activity towards a series of tested model substrates (Fig. 1d). In contrast to eukaryotic Pin representatives but similar to other prokaryotic parvulins studied so far [50, 51], archaeal PPIases do not catalyze the isomerization of peptides containing a phosphorylated serine residue preceding the proline. However, protein phosphorylation as a regulatory mechanism is not generally excluded as numerous genomes of archaeal organisms contain open reading frames for potential protein kinases and protein phosphatases in homology to known eukaryotic proteins [52]. The inability of *Nm*Pin to isomerize phosphorylated peptides is structurally reflected by the absence of a phosphate-binding domain or phosphate-binding protein extension, which is present in all known phospho-specific prolyl isomerases such as the WW-domain in human Pin1 and ESS1 from *C. albicans* [37, 53–55] or a distinct four-amino acid insertion in several plant representatives [56]. The phosphate recognition by *Nm*Pin becomes also very unlikely considering the predominantly negative surface potential around the active site due to the exposed Glu83, which is also involved in substrate binding as shown by NMR chemical shift perturbation experiments (Fig. 2a). The substrate selectivity of *Nm*Pin for amino acids with long side chains (Arg, Leu) preceding the proline moiety is mainly determined by the catalytic site Asp42. Despite the structural differences between the sdPar *Nm*Pin and the N-terminally extended human Par14, and the difference in the catalytic efficiency (Par14: *k*_*cat*_*/K*_*M*_ = 1.01 × 10^3^ M^–1^s^–1^ [57], *Nm*Pin: *k*_*cat*_*/K*_*M*_ = 5.66 × 10^5^ M^–1^s^–1^ for Suc-A-L-P-F-pNA), their substrate selectivity is nearly identical. This observation supports recent studies, where the TACK superphylum, including the Thaumarchaeota, were put in a phylogenetic sister relationship with Eukaryotes [58–61]. Although *Nm*Pin and Par14 seem to have the same substrate selectivity, their cellular localization seem to determine their different function in the cell since *Nm*Pin is important for the folding of extracellular proteins while the human Par14 is involved in signal transduction [62] and the maturation of ribosomal RNA [63].

### The surface layer provides *N. maritimus* cells with a rod-shaped structure

The vast majority of archaea possesses a single membrane and most of these membranes are covered by a protein layer, which consists of a single protein species often modified by glycosylation [49]. Our TEM data clearly indicate that the rod-shaped cells of *N. maritimus* are also enveloped by a surface layer (S-layer). The precise composition of this S-layer is unknown. However, upon stress, either mechanical (centrifugation) or osmotic (salt concentration), the cell shape is altered to a spheroidal form, which is accompanied by a ruptured S-layer (Fig 4c, f [35]). Additionally, some cells eject parts of their cytoplasm due to damage (Fig. 4h). Only a small number of cells are able to avoid the stress and remain intact. The S-layer maintains the cellular shape of the prokaryote. However, the exact physiological role of the S-layer in *N. maritimus* under natural conditions needs to be investigated. It is important for the shape of the organism but may also provide protection against natural enemies and viruses.

### *Nm*Pin is located on the outer membrane surface

The cytoplasmic membrane of *N. maritimus* consist of intact polar lipids with negatively charged phosphatidic, glycosidic or phosphoglycosidic head groups [43–45], which present an interface suitable for binding to the lysine-rich patch provided by *Nm*Pin. The use of eukaryotic lipids as a model system for our lipid sedimentation assays strengthens the pure electrostatic character of the *Nm*Pin-lipid interaction since no further anchoring seems to be required. This type of attaching PPIases to a membrane simply by electrostatic interactions might be a general feature of archaeal parvulins since the patch is also conserved in *Cs*PinA from *C. symbiosum*. In addition, the flat shape of the lysine cluster provides ideal conditions for an electrostatic membrane interaction, in contrast to DNA binding proteins, which mostly possess basic patches with significant curvature [64, 65]. A mutation of the highly conserved residue Lys7 [35] to Glu7 hampers binding of *Nm*Pin to lipids. For endogenously expressed *Nm*Pin, no integral lipid anchors or transmembrane helices for proper membrane binding were found. This is in contrast to the two known membrane-bound bacterial parvulins, where PrsA of *B. subtilis* is connected to the outer leaflet of the cell membrane with a lipid-anchor attached at an N-terminal cysteine residue [22, 29, 30] and PpiD of *E. coli*, which is embedded in the double-layer via an N-terminal transmembrane helix [31]. However, in human cells, several examples of peripheral membrane proteins are found where a nonspecific electrostatic interaction between a cluster of basic residues of the protein and acidic phospholipids in the membrane is required for activity and regulation [64]. In *N. maritimus* cells with an intact membrane, *Nm*Pin is observed and highly abundant on the surface (Fig. 4b, g). In contrast, the amount of *Nm*Pin in cells treated with PBS is significantly reduced. The hypoosmotic stress leads to a complete removal of the S-layer as well as a swelling of *N. maritimus* and, concomitantly, a different surface curvature which can interfere with lipid-*Nm*Pin complex formation. The multivalent negative phosphate ion, which is a strong competitor for the interaction with charged lipids [66], may have an additional effect. Both effects in combination could lead to the release of *Nm*Pin from the membrane under low salt conditions. Therefore, we assume that *Nm*Pin is located in the ‘quasi-periplasmic space’ between the membrane and the S-layer [67, 68] (Fig. 4i). Several ways for the secretion of folded and unfolded proteins in archaea have been reported. For example, the general secretion (Sec) pathway or the Twin arginine translocase (Tat) pathway that might also be responsible for the translocation of *Nm*Pin [69, 70].

**Fig. 5:**
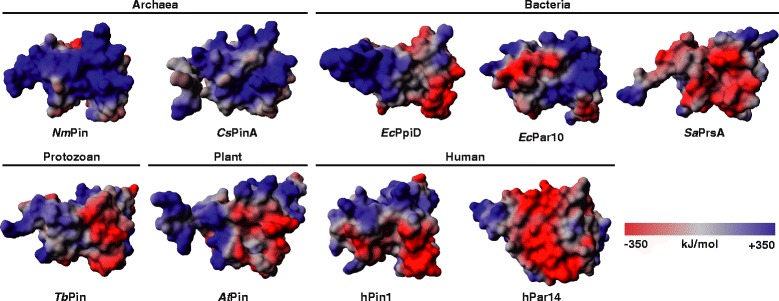
Electrostatic surface potential of various parvulin-type isomerases. Potentials were calculated with the Particle Mesh Ewald approach. The intensity of the potential is indicated by a color gradient from dark red (negative) over grey (neutral) to dark blue (positive) ranging from –350 to +350 kJ/mol. *Nm*Pin (*Nitrosopumilus maritimus*), *Cs*PinA (*Cenarchaeum symbiosum*), *Ec*PpiD (*Escherichia coli*), *Ec*Par10 (E*scherichia coli*), *Sa*PrsA (*Staphylococcus aureus*), *Tb*Pin (*Trypanosoma brucei*), *At*Pin (*Arabidopsis thaliana*), *h*Pin1 (human), *h*Par14 (human)

To investigate a potential general role of a lysine-patch for parvulins we calculated the electrostatic potential of the molecular surface of other homologues of *Nm*Pin (Table 2, Fig. 5). Interestingly, the positively charged lysine-patch seems to be conserved only in archaeal parvulins (*Nm*Pin, *Cs*Pin) while bacteria seem to use transmembrane helices or lipid anchors to attach their parvulins to membranes (*Ec*PpiD, *Sa*PrsA). No membrane attachment module (transmembrane helix, lipid anchor or lysine patch) can be found in higher eukaryotes. A membrane anchor or a transmembrane domain is perhaps a more advanced and safer way for organisms to attach a protein to a membrane and the membrane binding by a positively patch might be lost during evolution. To verify a conservation of the lysine-patch in Archaea we have done a sequence alignment of all archaeal *Nm*Pin homologues available from the NCBI database (Additional file 1). When comparing the positions of lysines contributing to the positively charged patch in *Cs*Pin and *Nm*Pin all Thaumarchaeota, Crenarchaeota and ARMANs may show a similar lysine-patch on their surface. However, parvulins from Euryarchaeota show only a weak conservation, which may lead to a lower affinity or even no binding to the membrane, while bacterial (*Ec*Par10) and eukaryotic parvulins (Par14, hPin1) lack this prominent feature completely, which is in line with electrostatic potential of the molecular surface of some representatives (Fig. 5). Hence, the lysine patch might be a general feature of parvulins from organisms of the TACK phylum rather than all Archaea.

### Functional role of *Nm*Pin

We monitored the localization of *Nm*Pin under different osmotic conditions and observed a strong accumulation of the isomerase next to the cell membrane under marine-like salt conditions (Fig. 4b, e). Under low salt stress, *Nm*Pin levels are significantly reduced (Fig. 4g), which contradicts an involvement in stress-related pathways and suggests a similar cellular role of *Nm*Pin as observed for the PPIases PrsA [23], PpiD [31] and SurA [19] in bacteria. The latter are located at the membrane and are involved in the maturation of extracellular, periplasmic and outer membrane proteins. We therefore assume *Nm*Pin to be involved in the folding of S-layer proteins and in maintaining the correct structure of the cell envelope. However, we do not yet have any information about the S-layer protein of *N. maritimus*. Nevertheless, our hypothesis of an pseudo-periplasmic space located PPIase is supported by the fact that the S-layer glycoprotein complex (tetrabrachion) of the related Crenachaeote *Staphylothermus marinus* contains a unique proline residue inside a V-I-P-K-F motif which separates the right-handed and left-handed supercoil parts [71, 72]. Obviously, the *cis-*/*trans*-conformation of the proline affects the structure of the glycoprotein complex and thereby influences the structure of the whole S-layer. Indeed, we could find a peptidylprolyl isomerase in *Staphylothermus marinus* with a high proportion of lysines (12.0 %) similar to *Nm*Pin (16.1 %) pointing towards a potential lysine patch for membrane binding whereby no transmembrane helices could be predicted. This indicates the importance of folding-assisting PPIases such as *Nm*Pin at the outside of the cell membrane for archaeal organisms.

## Conclusion

In this study, we determined the solution structure of the parvulin *Nm*Pin from the ubiquitous and ecological relevant thaumarchaeote *N. maritimus. Nm*Pin represents the first prolyl isomerase of the domain Archaea whose parvulin-like activity as well as membrane-associated location in vivo were characterized. Its structure revealed a lysine-rich patch, which was identified as a membrane-binding interface in vitro. In vivo *Nm*Pin is located at the outer surface of the membrane. The endogenous cellular expression level of the protein and its uniform distribution is highest in the presence of an intact cell envelope, precisely between the membrane and S-layer. Membrane association has been previously reported for multi-domain parvulins in bacteria [18, 21, 22] as well as in eukaryotes [73]. For *Nm*Pin we present a novel type of membrane association of a single-domain parvulin without any need for anchoring modifications or transmembrane domains, which might be a more general feature of archaeal parvulins since the same kind of membrane interaction was observed for *Cs*PinA. Considering recent studies, which suggest that the archaeal ancestors of eukaryotes are affiliated with the TACK superphylum, including the Thaumarchaeotes [58–61], *Nm*Pin likely represents a highly original member of the parvulin family. This assumption is supported by our results, showing that the *Nm*Pin folding topology and substrate selectivity are still conserved in the human Par14 protein [8, 57].

## Methods

### Cloning and mutagenesis

The *nmpin* gene was PCR-amplified with oligonucleotides forward (5′-CATTCGGGCCCTCAAACAAAATCAAATGTTCACAC-3′) and reverse (5′-TGCAGGGATCCTTATCCGAATCTCTTGATAATATG-3′) (Metabion) using genomic DNA of *N. maritimus* as a template. The resulting fragment containing the restriction sites for *Apa*I and *Bam*HI (NEB, Fermentas) was cloned into a modified pET-41b(+) (Addgene) vector as described elsewhere [74]. Mutants of *nmpin* were designed by site-directed mutagenesis using the QuikChange™ Lightning or the Q5 site-directed mutagenesis kit (Stratagene, NEB) and confirmed by Sanger sequencing (GATC GmbH).

### Protein expression and purification

For protein production, plasmids were transformed into *E. coli* BL21(TL3)T1r (Sigma). Unlabeled protein was produced in 1 L of 2 × YT medium, grown to an optical density at 600 nm (OD_600_) of 0.8 at 37 °C. For isotopically-labelled protein, cells of a culture of 1 L in LB medium (OD_600_ of 0.8) were transferred to 4 L M9 minimal medium supplemented with 1 g/L [^15^N]ammonium chloride and, if required, 4 g/L [^13^C]glucose and further grown to an OD_600_ of 1.0. Subsequently, protein expression was induced by addition of 0.2 mM IPTG and incubated overnight at 25 °C prior to centrifugation (3700 × *g*, 20 min, 4 °C). Cell lysis of the resuspended pellet in PBS at pH 8.0 was performed by sonification (Bandelin Sonopuls). Cell debris were removed by ultracentrifugation (95,800 × *g*, 4 °C, 60 min) and the supernatant was applied to a GSH-sepharose column (GE Healthcare) and eluted with 10 mM glutathione. The GST-tag was cleaved off by PreScission protease and the resulting *Nm*Pin protein purified by size exclusion chromatography (SEC) on a Superdex 75PG 26/600 (GE Healthcare) in 50 mM Tris and 150 mM NaCl at pH 8.0. Finally, the protein was dialyzed against buffers as indicated.

### MALDI-TOF

Recombinant *Nm*Pin (10 μg) was desalted with C18-tips (Supel-Tips C18, Supelco), eluted with 2 μL of 50 % (v/v) acetonitrile/0.1 % (v/v) trifluoroacetic acid and acidified with an equal volume of 2.0 % (v/v) trifluoroacetic acid. The sample was embedded in a saturated dihydroxyacetophenone matrix in ethanol with 25 % (v/v) aqueous ammonium citrate dibasic solution (18 mg/mL). Analysis was performed using an autoflex speed (Bruker) operated in a positive ionization and reflector mode. Spectra were recorded with flexcontrol and the dataset processed with flexanalysis (Bruker).

### *N. maritimus* cultivation and harvesting

*N. maritimus* cultivation was performed as described previously [35, 47, 75] in synthetic crenarchaeota media (SCM) with a starting concentration of 1 mM ammonium chloride as an energy source. Batches of 5 L were inoculated with 5 % (v/v) culture of *N. maritimus*, incubated at 29 °C in the dark without stirring and growth was monitored via nitrite concentration. Cells were harvested by centrifugation (4800 × *g*, 25 °C, 60 min) and pellets resuspended in PBS at pH 8.0.

### Western blot

α-*Nm*Pin antibody for detection of the endogenous protein was produced in rabbits with recombinant *Nm*Pin from *E. coli* as the antigen (Eurogentec). The final bleed was affinity purified against recombinant *Nm*Pin. For western blot analysis cells were washed in cold PBS at pH 8.0 with variable salt concentrations as indicated, supplemented with a protease inhibitor cocktail (Roche mini) and lysed by sonification (5 × 15 s at 60 % intensity, Sonopuls, Bandelin) prior to ultracentrifugation at 100,000 × *g*, 4 °C for 50 min. The supernatant was removed and the pellet resuspended in the same volume of PBS. Samples were subjected to SDS-PAGE and transferred by a semi-dry blot to a nitrocellulose membrane (30 min, 80 mA). Blocking of the membrane was done in PBST150 (PBS, pH 8.0, 150 mM NaCl, 0.1 % (v/v) Tween20) with 3 % milk powder at 4 °C overnight; 1:1000 diluted α-*Nm*Pin was incubated in the same buffer for 3 h at room temperature (RT). Washing of the membrane was done with one step PBST500 (PBS, pH 8.0, 500 mM NaCl, 0.1 % (v/v) Tween20) followed by two steps with PBST150, each 15 min at RT under shaking. Incubation of HRP-coupled α-rabbit IgG secondary antibody (Sigma) was done in a 1:2000 dilution in PBST150 with 3 % milk powder for 30 min at RT. The membrane was washed as before and subjected to chemoluminescent detection with SuperSignal West Femto Kit (Thermo) on a CL-XPosure film (Thermo).

### PPIase activity

Catalytic activity of *Nm*Pin was measured using a conformer-specific protease coupled assay as described previously [5, 76]. The chromogenic peptide substrates, following the scaffold Suc-Ala-Xaa-Pro-Phe-*para*nitroaniline (*p*NA), were pre-incubated overnight in 0.5 M LiCl in 2,2,2-trifluoroethanol at a concentration of 15 mM. α-chymotrypsin (35 μM; Sigma) was equilibrated with variable concentrations of *Nm*Pin for 5 min in PBS buffer at pH 6.8 at 10 °C. Subsequently, the assay was started by addition of 75 μM peptide and the reaction was spectrophotometrically monitored via *p*NA cleavage at 390 nm. For each substrate, the *cis*-content of the peptide and the reaction rate constant of chymotrypsin were determined on the basis of the uncatalyzed digestion. Using these constants the observed curves were fitted globally to a bi-exponential reaction equation with GraphPad Prism 5.04. The *k*_*cat*_*/K*_*M*_ was obtained from conditions with linear reaction rate to PPIase concentration correlation by using the following equation: *k*_*cat*_*/K*_*M*_ = (k_obs_-k_uncat_)/[PPIase].

### NMR spectroscopy and resonance assignment

Spectra were recorded on a Bruker Ultrashield 700 MHz spectrometer equipped with a cryoprobe unit at 300 K. For sample preparation, 1 mM *Nm*Pin was dissolved in 600 μL of 50 mM KP_i_ buffer, pH 6.5, 10 %/90 % (v/v) D_2_O/H_2_O or 100 % D_2_O containing 0.02 % (w/v) NaN_3_ and 50 μM DSS as calibration standard. Spectra were usually recorded using pulse sequences from the Bruker standard library (except ^1^H-^15^N-HSQC-NOESY). A set of a ^1^H-^15^N-HSQC, an HNCACB and a CBCACONH spectrum was sufficient to trace the chain of the protein sequence and to assign H_N_, N_H_, Cα and Cβ atoms of *Nm*Pin to their respective frequencies in the spectrum. The assignment of carbon atoms was completed using HCCH-TOCSY and -COSY spectra and the ^1^H assignment by ^1^H-^13^C- and ^1^H-^15^N-HSQC-TOCSY spectra. Aromatic hydrogen atoms were assigned by 2D spectra (TOCSY, COSY, NOESY in H_2_O and D_2_O). NOESY distance constraints were retrieved from 2D-NOESY and 3D ^1^H-^15^N-HSQC-NOESY spectra. Processing and evaluation of spectra was performed with Topspin 3.0 (Bruker), assignment was done with the CcpNmr-Analysis 2.3.1 software package [77].

### Structure calculation

NOE restraints were identified and transformed into distance constraints using the automated standard protocol of Cyana. For each hydrogen bond, retrieved from a series of ^1^H-^15^N-HSQC spectra after lyophilizing *Nm*Pin and dissolving it in 100 % D_2_O, two lower limit constraints were set for the distances from N to O and from HN to O. The structure of *Nm*Pin was calculated using Cyana 3.0 [78]. Owing to the high sequence identity (78.4 %) of *Nm*Pin to *Cs*PinA (PDB ID: 2RQS) a homology model was calculated using the software YASARA (Nova forcefield) and set as guide structure for a first cycle of calculation. Water refinement was performed with the software package YASARA using the structure module and the YASARA Nova forcefield [79]. Structural coordinates and NMR shift data were deposited in the RCSB databank (entry ID: 2MO8) and in the BMRB databank (entry ID: 18801), respectively.

### Chemical shift perturbation analysis

To a 200 μM sample of ^15^N-labelled *Nm*Pin in 50 mM KP_i_, pH 6.5, the peptide Suc-A-R-P-F-pNA in the same buffer was added stepwise to a final concentration of 15 mM. For each step, a ^1^H-^15^N- SOFAST-HMQC spectrum at 25 °C was recorded. All residues exhibiting chemical shifts ≥ 0.04 ppm were used to map the substrate binding interface on the surface of the *Nm*Pin structure using YASARA [36].

### Lipid sedimentation assays

Lipid-binding of *Nm*Pin_wt_, *Nm*Pin_K7E/K34E_ and *Nm*Pin_K7E_ was carried out as described previously with modifications [80, 81]. Brain lipid extracts from bovine (Folch fraction I, Sigma) were resuspended in HEPES buffer (20 mM HEPES, 150 mM NaCl, pH 7.4) to a concentration of 5 mg/mL under continuous stirring. The protein samples (15 μM) were incubated with varying liposome concentrations for 15 min at 37 °C and 350 rpm in a total volume of 40 μL and subsequently centrifuged (50 min, 100,000 × *g*, 4 °C). The supernatant was removed and the pellet resuspended in the equivalent volume HEPES buffer prior to analysis by SDS-PAGE.

### CD spectroscopy

CD spectra were measured in 0.1 cm cuvettes with 0.15 mg/mL protein in 50 mM NaP_i_, pH 7.4 at 20 °C as a sum of 50 single spectra with a Jasco J-710 spectropolarimeter (Jasco, Gross-Umstadt). A buffer baseline was subtracted from all datasets, units were converted to mean residue ellipticity and the mutant spectra normalized to the wildtype spectrum.

### Fluorescence microscopy

For localization of endogenous *Nm*Pin, 10 mL *N. maritimus* suspension in SCM medium, with or without addition of 4 % PFA, were centrifuged for 60 min at 4800 × *g* at 25 °C. The supernatant was discarded and the pellet resuspended in 200 μL SCM medium or PBS as indicated. Cells were incubated for 1 h at 29 °C for attachment to poly-lysine coated cover slips. Fixation was done with ice-cold 4 % PFA in PBS for 15 min at RT. For permeabilization, the PFA was supplemented with 0.1 % Triton. Five washing steps (each 3 min) with PBS were performed to stop the cross-link reaction. Blocking was done with 5 % goat serum in PBST (PBS, 0.1 % (v/v) Tween20) for 1 h at RT. α-NmPin antibody (1:50, rabbit) was incubated in 3 % goat serum/PBST overnight at 4 °C followed by four washing steps with PBST (each 3 min). Alexa488-coupled secondary α-rabbit IgG antibody from goat (Invitrogen) was diluted in 3 % goat serum/PBST (2 μg/mL) and incubated for 1 h at RT in the dark followed by four washing steps with PBST (each 3 min). DNA was stained with DAPI contained in the mounting medium (Vectashield, Vector Laboratories). Pictures were taken with a Zeiss Imager M2 with metal halide light source and the corresponding filter set (Alexa488: 495 nm/517 nm, exposure time: 500 ms, DAPI: 395 nm/461 nm, exposure time: 200 ns). Data processing was done with ZEN 2012 SP blue edition.

### TEM

Sample preparation for TEM microscopy was modified as described previously [82]; 10 mL *N. maritimus* suspension in SCM medium, with or without addition of 4 % PFA, were centrifuged for 60 min at 4800 × *g* at 25 °C. The supernatant was discarded and the pellet resuspended in 20 μL SCM medium or PBS as indicated for attachment to lacy carbon grids with ultrathin Formvar (200 nm mesh, Ladd Research Industries, Burlington, VT) for 30 min at RT. Cells were washed twice with SCM or PBS for 5 min at RT. Fixation was done with 2 % glutaraldehyde in PBS for 5 min at RT followed by three washing steps with PBS, 5 min each. Permeabilization was done with 2.5 % Triton in PBS for 5 min at RT followed by three washing steps with PBS, 5 min each. Blocking was performed with 5 % goat serum in PBST for 30 min at RT (three washing steps with PBST, 5 min each). α-NmPin antibody (1:20, rabbit) was incubated in 2 % goat serum/PBST for 3 h at RT followed by three washing steps with PBST, 5 min each. Secondary α-rabbit IgG antibody conjugated with 5 nm colloidal gold particles (1:20, goat, Sigma) in 2 % goat serum/PBST was incubated for 1 h at RT (three washing steps with PBST, 5 min each). The antigen-antibody complex was fixed again with 2 % glutaraldehyde in PBS for 5 min at RT followed by three washing steps in PBST, 5 min each. A final washing step was done for 15 min in ddH_2_O and subsequent air drying overnight. Pictures were taken with a JEOL 1400 plus (AMT UltraVUE camera) at 80 kV. Data processing was done with ImageJ.

## Additional file

Additional file 1: Figure S1. Sequence alignment of *Nm*Pin from *N. maritimus* with various homologues from other phyla to investigate a potential conservation of the positively charged lysine patch of *Nm*Pin. Representatives were found by BLAST search. Each group was separately aligned to *Nm*Pin (green). Lysines of the patch (K5, K7, K31, K34, K37, K47, K48, K90) are labelled in dark blue and Arg or positively charged residues which are in close proximity to the conserved position are labelled in light blue. The positively charged patch on the surface of *Nm*Pin might be conserved also in other members of the TACK phylum. (PDF 464 kb)
