# Supplementary material for: NmPin from the marine thaumarchaeote Nitrosopumilus maritimus is an active membrane associated prolyl isomerase
Source: BMC Biol. 2016 Jun 27;14:53. doi: 10.1186/s12915-016-0274-1 (PMC4922055; doi:10.1186/s12915-016-0274-1)
Supplement: Additional file 1: Figure S1. — Sequence alignment of NmPin from N. maritimus with various homologues from other phyla to investigate a potential conservation of the positively charged lysine patch of NmPin. Representatives were found by BLAST search. Each group was separately aligned to NmPin (green). Lysines of the patch (K5, K7, K31, K34, K37, K47, K48, K90) are labelled in dark blue and Arg or positively charged residues which are in close proximity to the conserved position are labelled in light blue. The positively charged patch on the surface of NmPin might be conserved also in other members of the TACK phylum. (PDF 464 kb) [file 12915_2016_274_MOESM1_ESM.pdf]

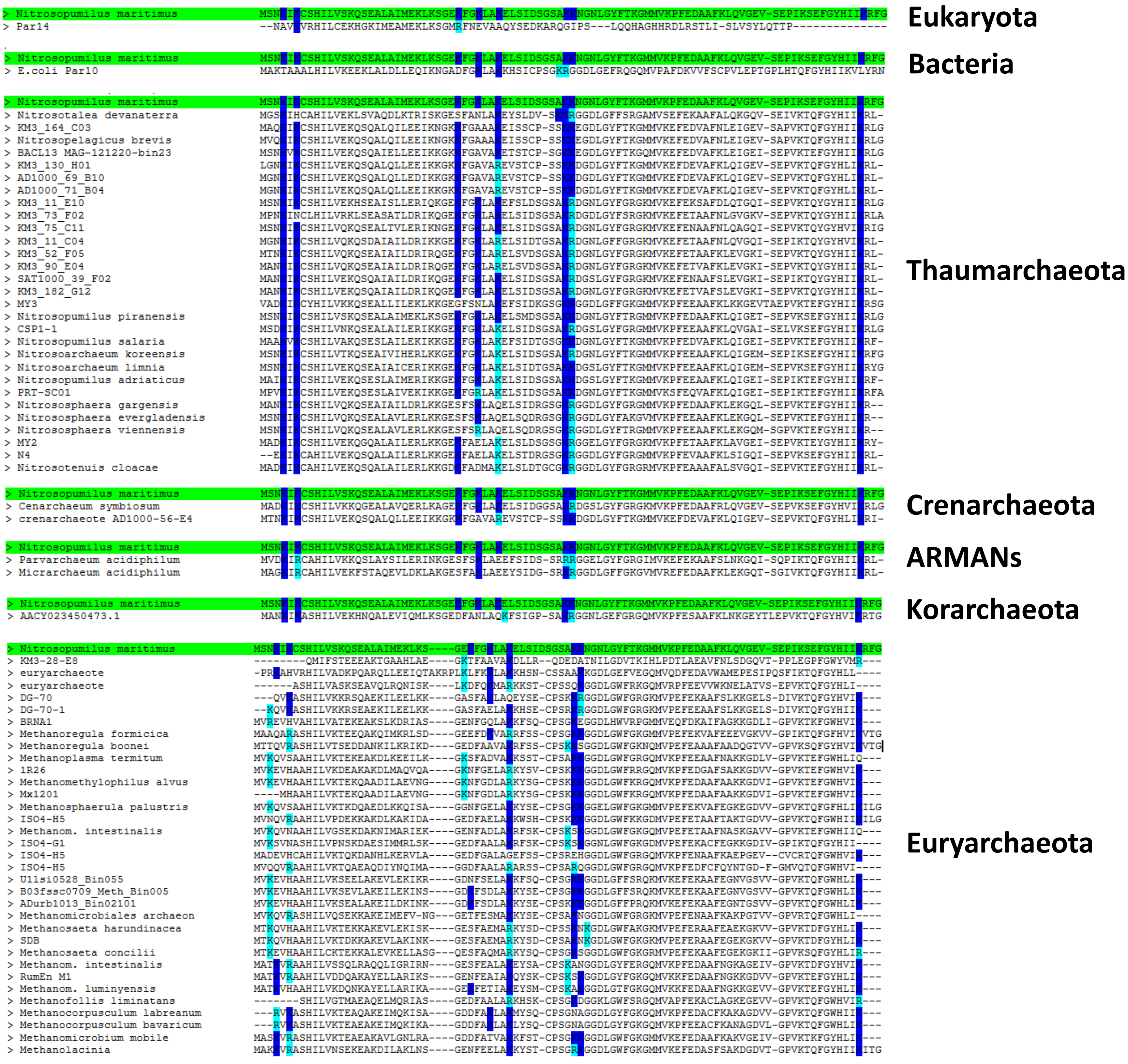
Eukaryota
Bacteria
Thaumarchaeota
Crenarchaeota
ARMANs
Korarchaeota
Euryarchaeota
> Nitrosopumilus maritimus
> Par14
> E.coli Par10
> Nitrosotalea devanaterra
> KM3_164_C03
> Nitrosopelagicus brevis
> BACL13 MAG-121220-bin23
> KM3_130_H01
> AD1000_69_B10
> AD1000_71_B04
> KM3_11_E10
> KM3_73_F02
> KM3_75_C11
> KM3_11_C04
> KM3_52_F05
> KM3_90_E04
> SAT1000_39_F02
> KM3_182_G12
> MY3
> Nitrosopumilus piranensis
> CSP1-1
> Nitrosopumilus salaria
> Nitrosoarchaeum koreensis
> Nitrosoarchaeum limnia
> Nitrosopumilus adriaticus
> PRT-SC01
> Nitrososphaera gargensis
> Nitrososphaera evergladensis
> Nitrososphaera viennensis
> MY2
> N4
> Nitrosotenuis cloacae
> Cenarchaeum symbiosum
> crenarchaeote AD1000-56-E4
> Parvarchaeum acidiphilum
> Micrarchaeum acidiphilum
> AACY023450473.1
> KM3-28-E8
> euryarchaeote
> euryarchaeote
> DG-70
> DG-70-1
> BRNA1
> Methanoregula formicica
> Methanoregula boonei
> Methanoplasma termitum
> 1R26
> Methanomethylophilus alvus
> Mx1201
> Methanosphaerula palustris
> ISO4-H5
> Methanom. intestinalis
> ISO4-G1
> ISO4-H5
> ISO4-H5
> U11si0528_Bin055
> B03fssc0709_Meth_Bin005
> ADurb1013_Bin02101
> Methanomicrobiales archaeon
> Methanosaeta harundinacea
> SDB
> Methanosaeta concilii
> Methanom. intestinalis
> RumEn M1
> Methanom. luminyensis
> Methanofollis liminatans
> Methanocorpusculum labreanum
> Methanocorpusculum bavaricum
> Methanomicrobium mobile
> Methanolacinia
